# Qualitative study on the evaluation of humanistic care quality in geriatric nursing based on three-dimensional quality structure theory

**DOI:** 10.3389/fmed.2025.1678646

**Published:** 2025-10-24

**Authors:** Jinrong Yuan, Ling Li, Qin Zhan, Huijuan Liu, Yilan Liu, Yanjie You

**Affiliations:** Department of General Medicine, Union Hospital, Tongji Medical College, Huazhong University of Science and Technology, Wuhan, Hubei, China

**Keywords:** human caring, three-dimensional structure, geriatric nursing, older adult, qualitative research

## Abstract

**Aims:**

To understand the current situation of nursing humanistic care in geriatrics, to integrate the humanistic care quality evaluation viewpoints based on the Donabedian’s three-dimensional quality structure theory, and to provide a reference direction for constructing a nursing humanistic care quality evaluation system in geriatrics specialties.

**Methods:**

4 geriatric nursing managers and 8 geriatric nurses of our hospital were selected for semi-structured interviews from October 2024 to December 2024, and based on the content analysis method, the data were analyzed and themes were refined using NVivo12 software.

**Results:**

A total of 12 sub themes were extracted. The structural level: rationalization of human resources, caring hospital environment, system and responsibilities; Process level: caring communication, identifying individual needs, patient medication safety, prevention of high-risk patients, medical and nursing cooperation, and humanistic literacy of nursing personnel; Outcome level: satisfaction, patient outcomes, and incidence of adverse outcomes.

**Conclusion:**

The quality of geriatric nursing humanistic care is still room for improvement. We further optimize the structure of human resources by allocating psychiatric nurses and constructing a stratified nursing response mechanism to satisfy personalized humanistic care, and encourage the participation of family members to ensure the continuity of patient care, and ultimately improve the information-based nursing system to realize the full coverage of humanistic care and the transformation of geriatric nursing from “disease-oriented” to “whole-person care.”

## Introduction

Currently, the global trend of population aging is intensifying. Data from the World Health Organization indicates that by 2023 (1), the proportion of the population aged 60 years and older exceeded 20%, and it is projected that by 2040, this proportion will reach 28% in China. As one of the countries experiencing the most rapid aging (2), China has seen geriatrics develop rapidly as a key discipline to address the challenges of an aging society. However, the geriatric department in China started relatively late and faces challenges including a shortage of specialized nursing human resources, uneven distribution of medical resources, and insufficient multidisciplinary collaboration (3). Concurrently, hospitalized older adult patients encounter multiple challenges. Physiological functional decline leads to mobility impairments, cognitive disorders, and polypharmacy risks, significantly increasing the complexity of nursing care (4). Furthermore, studies reveal that the prevalence of depression and anxiety among hospitalized older adults is considerably higher than in the general adult population (5). Additionally, traditional nursing models often inadequately address the individualized needs of older patients, further exacerbating adverse outcomes; for instance, the incidence of complications such as pressure injuries and delirium increases by 15–20% (6).

Consequently, given the limitations of traditional nursing models in addressing the complex needs of older patients, humanistic care has emerged as a critical direction for optimizing nursing practice in recent years (1). In this context, humanistic care specifically refers to nursing practices that fulfill patients’ psychological, social, and cultural needs through personalized communication, environmental adaptations, and family-involved decision-making (7). Older patients exhibit a particularly urgent need for humanistic care due to their unique physiological and psychological characteristics. However, geriatric nurses in China often demonstrate underdeveloped humanistic care competencies, compounded by a lack of standardized training programs (8). China’s “14th Five-Year Plan for Healthy Aging” explicitly calls for “building an elderly-friendly healthcare environment” (9), further underscoring the imperative for localized humanistic care models in geriatric settings. Nevertheless, despite widespread recognition of its importance, the implementation effectiveness of humanistic care in geriatric nursing remains inadequately evaluated due to the absence of standardized assessment tools, making it difficult to comprehensively reflect the quality of care delivered.

Donabedian’s three-dimensional quality framework provides the foundational model for evaluating healthcare service quality (10). This theory deconstructs healthcare quality into three interrelated dimensions: The “Structure dimension” refers to the foundational conditions for service delivery, encompassing facility and equipment configuration, staffing allocation, and organizational resources. The “Process dimension” focuses on the implementation activities involved in delivering healthcare services. The “Outcome dimension” pertains to the end results or consequences of the healthcare services provided. These dimensions are inherently linked: optimal structure lays the groundwork for standardized processes, which in turn directly contribute to achieving desired outcomes, forming a closed-loop system for continuous quality improvement. Currently, the Structure-Process-Outcome framework has been successfully applied across diverse clinical settings, including Intensive Care Units and emergency resuscitation, demonstrably enhancing nursing quality within these domains (11). The Structure-Process-Outcome theory offers distinct advantages for nursing quality evaluation and improvement: Firstly, it transcends traditional outcome-only evaluation models by establishing a systematic quality analysis pathway through the tripartite linkage of structure, process, and outcome. Secondly, it provides an evidence-based approach for optimizing quality improvement initiatives by clarifying the causal pathways. Lastly, this framework effectively addresses the challenge of measuring the abstract concept of humanistic care, enabling its systematic assessment within the quality paradigm (12).

In the face of the urgent need for nursing care quality improvement in geriatrics, the three-dimensional quality structure theory provides an ideal framework for constructing a scientific, systematic, and humanized humanistic care quality evaluation system. The integrated application of this theory will empower geriatric care quality improvement in all aspects from system construction to clinical practice (13). Therefore, based on the three-dimensional quality structure theory (10), this study was conducted through semi-structured interviews to understand the current situation of nursing humanistic care in geriatrics and the viewpoints of humanistic care quality evaluation, to provide a reference direction for the construction of a nursing humanistic care quality evaluation system for geriatrics specialties, so as to quantify the effectiveness of humanistic care and provide a basis for the optimization of service processes.

## Methods

### Study design

This study was based on Donabedian’s Structure-Process-Outcome framework. Aligned with the research objectives, the final interview guides were developed through a rigorous process: a comprehensive review of domestic and international literature; extensive discussions among the research team members; and consultation with relevant experts. The guides were subsequently refined and finalized following preliminary interviews conducted to test their clarity and appropriateness.

Nursing manager interview outline: ① Please talk about the current situation of the quality of humanistic care for geriatric patients? ② Do you think that humanistic care in geriatrics is different from other departments? In what aspects? ③What aspects do you emphasize when supervising the quality of nursing humanistic care? What is the basis? ④ If you were asked to develop evaluation indicators for the quality of humanistic care for geriatric patients, what would you focus on? Please talk about your ideas from the structural level (i.e., human resources, ward environment, etc.), the process level (i.e., the process of implementing humanistic care), and the outcome level (i.e., patient outcomes, satisfaction, etc.).

Nursing workers interview outline: ① Please talk about the current situation of the quality of humanistic care for patient care in the geriatrics department? ② Do you think humanistic care in geriatrics is different from other departments? In what aspects? ③ What are the main care needs of the geriatric patients you have taken care of? and ④ What aspects do you think should be reflected in the quality evaluation content of humanistic care in geriatric patient care? Please talk about your ideas from the structural level (i.e., human resources, ward environment, etc.), the process level (i.e., the process of implementing humanistic care), and the outcome level (i.e., patient outcomes, satisfaction, etc.).

### Participants and setting

Purposive sampling method was used to select nursing managers and nursing staff of the geriatrics department of our hospital from November to December 2024 as the interview subjects. Inclusion criteria: nursing managers: ① title of supervisor nurse or above; ② more than 5 years of working experience in geriatric department; ③ more than 3 years of nursing management; and ④ patients who voluntarily participated in this study; nursing staff: ① title of nurse or above; ② working in geriatric department for 2 years or above; and ③ patients who voluntarily participated in this study. After interviewing three nursing managers and six nursing staff, the data reached saturation, and then one nursing manager and two nursing staff were included to determine that no new topics appeared. 4 nursing managers, numbered M1 ~ M4, were finally included, and their general information is shown in Table 1; 8 nursing staff, numbered N1 ~ N8, and their general information is shown in Table 2. The study subjects gave informed consent to voluntarily participate in this study.

**Table 1:** Main characteristics of geriatric nursing managers.

Participants	Age	Gender	Title	Years of experience in nursing management work	Years of experience in geriatric nursing
M1	43	Female	Supervisor nurse	10	20
M2	37	Female	Supervisor nurse	3	9
M3	44	Female	Supervisor nurse	10	20
M4	40	Female	Supervisor nurse	7	18

**Table 2:** Main characteristics of geriatric nurses.

Participants	Age	Gender	Title	Years of working
N1	41	Female	Supervisor nurse	22
N2	26	Female	Primary nurse	4
N3	40	Female	Supervisor nurse	20
N4	27	Female	Primary nurse	2
N5	25	Female	Primary nurse	3
N6	30	Female	Supervisor nurse	8
N7	36	Female	Supervisor nurse	15
N8	35	Female	Supervisor nurse	12

### Data collection

A semi-structured interview method was used. The researchers were all geriatric nurses with graduate education in our hospital, and received unified training to establish a friendly relationship with patients before the interview. A pre interview was conducted before the formal interview to investigate whether the researchers’ interview methods and contents were correct, and the interview outline was further optimized. During the formal interview, relatively quiet environment was chosen for face-to-face interviews, which lasted about 20–30 min, and were recorded after obtaining the interviewer’s consent. The respondents were informed that they had the right to terminate or withdraw from the interview during the interview, and the researcher needed to maintain a neutral attitude and flexibly adjust the interview questions. The researcher needs to transcribe the audio-recorded data into textual data in a timely manner and check with the interviewees to ensure the authenticity and accuracy of the data.

### Data analysis

This study used NVivo12 software for content analysis and theme refinement (14). Within 24 h after the interviews, the recordings were converted into text, and the initial coding and categorization was conducted independently by 2 researchers, with reference to Donabedian three-dimensional theory to guide the categorization of themes, after the classification was completed, the final theme was formed through discussion and confirmation, and a total of 3 themes and 11 sub themes were extracted.

### Trustworthiness

The interview process followed the principles of fairness, impartiality and respect, and the content and timing of the interview outline was adjusted in a timely manner according to the content of the interview. The content of the interviews was expressed in easy-to-understand terms, and the interviews were confirmed over and over again to ensure the accuracy of the interviews. After interviewing until no new themes emerged, the researcher interviewed three interviewees who met the inclusion criteria again to ensure the scientific nature of the interviews.

### Ethical considerations

This study received ethical approval from the Medical Ethics Committee of Union Hospital, Tongji Medical College of Huazhong University of Science and Technology, China, under the reference Ethics (Approval No. 0312). The researchers provided a detailed explanation of the study’s objectives to the participants, ensuring their understanding of the research purpose.

## Results

After data analysis, 12 subcategories were extracted related to threats to the conversations. Each of these categories includes subcategories, which are summarized in Table 3.

**Table 3:** Theme, main categories and subcategories related to humanistic care quality in geriatric nurses.

Main categories	Theme
Structural level	Rationalization of human resources
	Caring hospitalization environment
	System and responsibilities
Process level	Caring communication
	Meeting individual needs
	Patient Medication Safety
	Prevention of high patient risk
	Medical and nursing cooperation
	Humanistic quality of nursing personnel
Outcome level	Satisfaction
	Patient outcomes
	Incidence of adverse outcomes

### Structural level

#### Rationalization of human resources

Reasonable human resources can provide a good foundation for the effective implementation of humanistic care. A reasonable bed nurse ratio is conducive to the implementation of nurses’ humanistic care. Through the training of psychological specialist nurses and geriatric specialist nurses, professional humanistic care guidance for the elderly should be one of the evaluation criteria of humanistic care.

M4: “Now the bed-to-nurse ratio in our department is reasonable, which facilitates the nurses to have more time and energy for caring for each patient.” N1: “I think the ratio of high, middle and low level nurses is important because the middle and high level nurses will lead by example and the low level and trained nurses will learn from their way of caring and do a good role model.” M2: “Although our nurses have done more comprehensive humanistic care, there are still places to fill, we nurses are limited in our ability to care psychologically, but psychological knowledge is complex, and having psychologically qualified nurses would be more conducive to caring psychologically for depressed and anxious older patients.” M3: “After all, we have not received systematic humanistic care training, if there is a geriatric specialist nurse in the department, it will make the patients feel the professionalism of the nursing team, which is conducive to the implementation of care.”

#### Caring hospitalization environment

The interviewees said that the elderly’s body function is degraded, and the composition of the environment needs to provide convenience for the elderly and create a humanistic atmosphere. Through the optimization of the physical environment and the shaping of the humanistic environment, the elderly patients’ feelings of care in life and mind can be increased. At the same time, some respondents mentioned the importance of informatization in the implementation of humanistic care.

M1: “I believe that the evaluation of the quality of humanistic care in geriatrics should focus on the humanistic environment, for example, the wall posters of the common chronic diseases of the elderly of educational posters, posters on prevention of deep vein thrombosis and prevention of falls, etc.; at the same time, older people’s ability to understand is relatively poor, so we post obvious and easy-to-understand examination guideline sheets, which shapes the humanistic environment of the department.” N8: “We have L-shaped handrails in our toilets, which are safe for tired and weak elderly people who can get up with the help of this handrail when they get up from the toilet.” M2: “We provide public wheelchairs, crutches, etc. for inpatients who have difficulty with their legs and feet to go down for checkups.” N4: “I think it is important to create a humanistic atmosphere. For example, we will play health education videos to patients in the afternoon, which not only enriches the hospitalized life of elderly patients, but also improves their awareness of diseases.” M3: “I hope that the department will realize information technology soon, which will not only improve our work efficiency, but also make care plans for patients in a comprehensive way and provide humanistic care in a targeted way.”

#### System and responsibilities

Interviewees mentioned that at the system level, the establishment of a perfect geriatric specialty humanistic care system, nursing management system, nurse incentive system and clear job duties and personnel division of labor can lay a very important foundation for the establishment of the geriatric department’s humanistic care standardization.

M3: “So far, there is no geriatric specialty-specific humanistic care system in China, and if we can establish a perfect system, nursing workers’ humanistic care for geriatric patients is more standardized and has reference significance.” N2: “In addition to the necessary nursing management system, I think we also need to improve the incentive system for nurses, such as the monthly selection of caring stars and other initiatives to motivate nurses to improve their humanistic caring ability.” M4: “We have clear requirements for the duties of each nursing position, and in addition to this, a reasonable division of personnel, such as the health promotion group, the psychological care group, the wound care group, etc., is carried out to provide all-round humanistic care for the elderly.”

### Process level

#### Caring communication

Communication is a central pillar in building a humanistic patient care practice, and its importance is particularly emphasized in the field of geriatric care. Elderly patients are commonly experiencing age-related physiologic decline, and these physiologic changes significantly increase the complexity and challenge of communication, making conventional methods of information transfer often ineffective. In this context, “caring communication” goes beyond the mere exchange of information and becomes a central vehicle for conveying respect, understanding, empathy and building trust. For elderly patients, effective caring communication reflects the nursing staff’s high professionalism and humanistic feelings.

N6: “In our daily nursing work, we explain the operation items and purposes of each patient, including those who have been bedridden for a long period of time and whose consciousness is blurred, before the nursing operation, which is a sign of respect for them.” N7: “We have a good sense of respect for our patients, including those who have been bedridden for a long period of time and whose consciousness is blurred.” N3: “We are very patient in communicating with elderly patients, not interrupting them and listening patiently to queries. For example, if elderly people find it difficult to understand the process and method of payment, we will also inform them patiently and help them in time.” N2: “What makes our geriatrics department different from other departments is that we help all patients to make appointments for examinations and inform them at the bedside, explaining in detail the precautions to be taken, and on the day of the examination we also remind them of what they are going to have to do, which demonstrates good caring communication in this process.”

#### Identify individual needs

Interviewees said that humanistic care for older patients is not a one-size-fits-all service, but rather a “customized” practice based on the patient’s unique life story, physical condition, psychological state, and social relationships. The core of achieving quality humanistic care for elderly patients lies in accurately identifying and effectively meeting their multidimensional and individualized needs. Interviewees emphasized the importance of going beyond standardized care processes and making “personalization” the starting and ending point of care practices. From the perspective of staff, in addition to meeting the personal requirements of patients, high-quality humanistic care needs to identify the individual needs of elderly patients with different conditions, and emphasize the ability to actively identify and optimize the needs assessment of different individuals.

N1: “We will focus on the physiological individual needs of the elderly. For example, for patients with high fever, we take the initiative to check if their clothes are wet with sweat every hour and send them new gowns; for patients who are hard of hearing, we will approach them to amplify the volume of their speech so that they will not be belittled.” M1: “We pay a lot of attention to the sleep of elderly patients; for patients with insomnia, we pay attention to the night rounds and counsel them on sleep aids.” N4: “For patients with cognitive impairment, we regularly exercise their cognitive abilities rather than allowing them to deteriorate; the charge nurse will actively observe and promptly identify older adults with anxiety and depression, carefully hand over the shift, and intervene.” N6: “We focus on continuity of care and social support for older adults, encouraging patients to participate in activities after discharge during chats and educating patients’ families about the disease, which facilitates joint disease management after discharge.”

#### Patient medication safety

Respondents’ emphasized that medication safety is no longer a purely pharmacological or technical issue, but is deeply embedded in the core dimensions of humanistic care practice. True humanistic care necessarily involves a deep understanding of the fragile pharmacological state of elderly patients and a strong commitment to safeguarding them from medication harm. The systematic assessment and continuous improvement of medication safety as a hard and fast indicator of quality evaluation of humanistic care in geriatrics not only significantly reduces medical risks and improves patient outcomes, but also guards the last line of defense for the dignity and quality of life of geriatric patients.

M2: “Nursing staff should ensure that medication is dispensed to the hand and taken to the mouth, and that medication is dispensed in a timely manner. If the patient is not present, a strict handover is required to ensure the safety of the patient’s medication.” M4: “I think nursing staff should be familiar with the contraindications of drugs; at the same time, the drip rate of infusion for elderly patients needs to be strictly controlled to ensure the comfort and safety of patients during infusion.”

#### Prevention of high patient risk

Nursing staff will proactively and timely identify and deal with the high risks that exist in patients to prevent the occurrence of adverse nursing events and ensure patient safety.

N4: “There is a bed of grandpa who is bed-ridden for a long time and has dermatitis, we will carefully observe his skin during the shift handover and on the duty nursing shift, with pressure redness and immediately use decompression patch to protect it, and with the unavoidable rupture, we will take the initiative to use the growth factor to apply it and protect it with ulcer patches instead of waiting until the doctor notices it to start treatment.” N8: “Our nurses are very friendly and always tell patients at high risk for falls to be careful when walking and not to fall, and patients at high risk for blood clots to move their legs when lying in bed to prevent clots from forming.”

#### Medical and nursing cooperation

Interviewees indicated that the harmony and good cooperation between healthcare professionals and nurses could facilitate the implementation of humanistic care in the geriatric department and enhance the atmosphere of humanistic care. This collaboration is essentially a process of building an interprofessional community of care that is “centered on the elderly patient,” and its efficacy directly determines the depth of the translation of humanistic care from concept to practice.

N3: “For patients who are critically ill, seriously ill, or have an acute onset of illness, we will communicate with the doctors in a timely manner and pay attention to the changes in their conditions, so that we can better deal with the patients’ conditions.” M3: “At present, doctors’ humanistic care behavior is weaker than ours, which sometimes hinders the full implementation of humanistic care. Therefore, we should also instill the concept of humanistic care into doctors, strengthen medical cooperation and doctors’ humanistic care ability, and improve patients’ satisfaction.”

#### Humanistic quality of nursing personnel

The humanistic quality of nursing staff is enhanced and standardized through humanistic care training and sharing of caring examples in the hospital, district and department. Through systematic training, special learning and index evaluation, we can optimize the measures of humanistic care.

M4: “We have diversified humanistic care training modes for nursing staff, such as online teaching, offline demonstration, and reading of relevant books, etc., and the humanistic care literacy of nursing staff and nursing managers has been enhanced a lot.” M1: “We share humanistic care examples every month, which not only makes nursing staff focus on caring for patients, but also learn by ear and thus apply it to their work.”

### Outcome level

#### Satisfaction

Satisfaction is not only the intuitive embodiment of patients’ evaluation of nursing workers’ humanistic care behavior and nursing work, but also the direct indicator of humanistic care quality evaluation.

N7: “For us, the satisfaction of patients and patients’ families must be what we are most concerned about, which is the most direct reflection.” M2: “I think that the evaluation of nursing humanistic care is not only on the side of patients, but also the evaluation of the satisfaction of nursing staff themselves as well as the evaluation of doctors’ satisfaction with nursing humanistic care work.”

#### Patient outcomes

Interviews showed that whether patients were discharged better than before admission, and whether psychological and social support aspects were improved was one of the important indicators for evaluating humanistic care.

N7: “Whether the patient’s depression and anxiety are reduced, cognition is better, and daily living ability is improved before discharge are all issues that need to be focused on after treatment and care, rather than treating the symptoms.” M4: “We hope that the hospitalized elderly, after treatment in our unit and through the guidance of the nurses, have learned more knowledge related to the disease and become more skillful and correct in subsequent home medication and care.” M1: “We hope that through humanistic care, we can make the hospitalized patients pay attention to their illnesses, but they should not be overly worried, and how to balance the patient’s emotions and have a healthy psychological state when they are discharged from the hospital is also very important.”

#### Incidence of adverse outcomes

Elderly patients have more high-risk factors, and adverse outcomes may occur if care is not appropriate or if less attention is paid to the patient. Therefore, interviewees believed that the incidence of adverse outcomes could be used as one of the outcome evaluation indicators.

M2: “Due to the special characteristics of geriatric patients, we have to strictly prevent the occurrence of adverse outcomes such as falls, unplanned extubation, and pressure injuries, all of which require less attention and care from nursing staff.” M3: “Safe management of medication in the elderly is very important and takes a lot of caregiver’s time to ensure the safety of medication; therefore, I believe that the rate of medication administration errors is also an important indicator of adverse outcomes.”

## Discussion

### Current status of humanistic care in geriatric nursing

This study reveals that while humanistic care practices in geriatric nursing in China have made progress across the Structure, Process, and Outcome dimensions, challenges related to systematic inadequacies persist.

#### Rationalization of nursing staffing and cultivation of a humanistic care environment

Within the Structure dimension, the allocation of nursing human resources in geriatric departments is gradually becoming more rational. An appropriate nurse-to-patient ratio and the deployment of geriatric specialist nurses lay the foundation for humanistic care delivery (15). With the establishment of the Department psychological team, the allocation of psychological specialist nurses is relatively low, and it is unable to provide deeper and more professional humanistic care for the elderly, which is still far from the International Humanistic Care model (16). Furthermore, this study indicates that age-friendly modifications to the inpatient environment-such as the provision of mobility aids and tailored educational guidance-help create a warm atmosphere perceived by patients, significantly enhancing their sense of security and convenience (17). Given the prevalence of frailty, disability, and cognitive impairment among older adults, fostering such a supportive inpatient environment can effectively prevent or reduce adverse events like falls, wandering, and injuries during hospitalization, this is consistent with previous research conclusions (18).

#### Multidimensional enhancement of the humanistic care process

Within the Process dimension, nursing staff demonstrate a patient-centered philosophy through practices such as patient communication, identifying individualized needs, and implementing high-risk prevention strategies (19). Specific measures to ensure medication safety effectively reduce errors associated with polypharmacy in older patients (20). Concurrently, nursing managers have enhanced staff competencies through sharing humanistic care exemplars and offering targeted training courses. However, standardized training programs aimed at systematically cultivating humanistic literacy require further development (21). However, at present, the training of humanistic care has not been standardized in China, resulting in differences between departments and hospitals, and it is difficult to unify the evaluation criteria.

#### Moving beyond traditional satisfaction: enriching outcome metrics

Within the Outcome dimension, this study moves beyond the limitations of traditional satisfaction surveys by integrating patient-reported outcomes and multidimensional adverse event rates. These findings align closely with initiatives like Comprehensive Geriatric Assessment (CGA), which aim to reduce adverse events and improve quality of life (22).

### Strategies to enhance humanistic care quality in geriatric nursing

#### Optimize staffing allocation and enhance professional expertise

This study highlights that a shortage of psychologically trained nurses continues to limit the depth of psychological support (23). Therefore, increasing the proportion of both geriatric specialist nurses and psychologically trained nurses is crucial. Geriatric specialist nurses, equipped through systematic training with core competencies in managing geriatric syndromes and multimorbidity care, significantly elevate the quality of nursing services (24). Research demonstrates that units staffed with geriatric specialist nurses achieve a 23% reduction in fall rates and an 18% increase in patient satisfaction (25). Introducing psychologically trained nurses addresses the gap in psychological support, employing evidence-based methods to alleviate anxiety and depression. We recommend healthcare institutions establish dual-certification programs in geriatric nursing and psychological care, incorporating these credentials into promotion evaluation systems to incentivize professional development.

#### Develop tiered care pathways to meet individualized needs

Since geriatric patients have different conditions, patients can be categorized into basic needs layer, intervention needs layer and interdisciplinary needs layer through comprehensive assessment, and different degrees and ways of care can be given to patients with different needs (26). For patients with good self-care ability, we mainly provide environmental adaptive care, such as providing a quiet and comfortable inpatient environment, so that patients can quickly adapt to inpatient life; for patients with moderate dysfunction, we implement symptomatic intervention care, such as proactively paying attention to the patient’s condition, and discovering and adopting nursing interventions before the patient proposes discomfort, etc.; for patients with complex health problems and those who are seriously ill, it is necessary to establish an interdisciplinary team composed of nursing experts, doctors, dietitians, rehabilitators, etc., so that patients and their families can feel respect and improve the quality of life of patients (27). At the same time, continuous quality improvement measures should be taken to improve patient satisfaction.

#### Promote patient and family engagement in care decisions and ensure continuity

Active engagement of patients and families is central to humanistic care. Nurses should facilitate shared decision-making, respecting patient values and preferences. This includes transparently discussing the risks/benefits of care plans, disease management, and potential medication side effects (28). Family involvement significantly reduces loneliness and improves treatment adherence in older patients (29). Encouraging families to participate in health education and supporting post-discharge management through instruction in home care skills and psychological support techniques is vital.

#### Innovate IT-enabled support systems for comprehensive humanistic care

This study identifies information technology (IT) as a highly anticipated enhancement by nursing staff. IT offers efficient tools for humanistic care, such as utilizing AI or robotics to analyze patient emotional states and provide early warnings for psychological crises (30); developing intelligent medication safety monitoring systems that integrate electronic health record data with drug interaction databases to minimize potential medication errors (30). These innovations can significantly improve the quality and reach of humanistic nursing care.

## Summary

In this paper, through semi-structured interviews with patients, nursing staff and nursing managers in geriatric wards, we discuss the viewpoints of humanistic care quality evaluation in geriatric nursing on the basis of three-dimensional quality structure theory, which is of some reference value for the construction of the subsequent system. However, this study still has some limitations. Firstly, the sample size is small and concentrated in a single medical institution, which may limit the generalizability of the results. However, although the interview was conducted in the same hospital, the Department of geriatrics in our hospital has a total of five floors, each floor has the same humanistic care for the elderly, but also has different implementation norms and priorities, which is also saturated for the research data and relatively reliable; secondly, the interview method relies on subjective expressions and is susceptible to recall bias, and in the future, the reliability and validity of the indexes need to be further verified through multi-center and large-sample studies, so as to promote the standardization of the training of nursing humanistic care, strengthen the psychological knowledge and communication skills of nursing staff, and at the same time, facilitate the conduct of longitudinal based on the findings of the present study. At the same time, based on the findings of this study, longitudinal studies can be conducted to track the long-term effects of humanistic care, and through multidimensional improvement, it is expected to realize the transformation of geriatric care from “disease-centered” to “whole-person care”, which will ultimately improve the quality of life of elderly patients and the social benefits of the healthcare system.

## Data Availability

The original contributions presented in the study are included in the article/supplementary material, further inquiries can be directed to the corresponding authors.
